# Prognostic Value of the Richmond Agitation-Sedation Scale for Mortality and Admission in Older Emergency Department Patients

**DOI:** 10.3390/medicina62091803

**Published:** 2026-09-19

**Authors:** Secdegül Coşkun Yaş, Mehmet Ali Aslaner

**Affiliations:** Department of Emergency Medicine, Faculty of Medicine, Gazi University, Ankara 06560, Türkiye; maliaslaner@gazi.edu.tr

**Keywords:** Richmond Agitation-Sedation Scale, Glasgow Coma Scale, older adults, emergency department, arousal, consciousness, hospital mortality, retrospective studies, cohort studies, prognosis

## Abstract

*Background and Objectives:* Altered arousal may provide rapid prognostic information in older emergency department (ED) patients. Originally developed to monitor agitation and sedation in critically ill patients, the Richmond Agitation-Sedation Scale (RASS) provides a rapid measure of arousal. However, its prognostic value relative to the Glasgow Coma Scale (GCS) in older ED patients remains uncertain. We evaluated whether RASS was associated with clinical outcomes and provided information beyond GCS. *Materials and Methods:* This study was a single-center retrospective cohort study of nontraumatic ED patients aged ≥ 65 years from January 2024 through October 2025. Only the first ED visit per patient was included. The primary outcome was in-hospital mortality; secondary outcomes were hospital and intensive care unit (ICU) admission. RASS was modeled categorically to preserve deviations in both directions from 0. Multivariable logistic regression and DeLong AUC comparisons were performed. *Results:* Among 10,208 patients, 352 (3.4%) died, 2301 (22.5%) were hospitalized, and 856 (8.4%) were admitted to the ICU. Mortality increased from 2.2% at RASS 0 to 59.4% at −5 and 45.5% at +3. Hospital and ICU admission rates also increased in both directions from RASS 0 (all trend *p* < 0.001). After adjustment for age, sex, vital signs, and GCS, both reduced arousal (RASS ≤ −4: aOR, 4.93; 95% CI, 2.10–11.61) and agitation (RASS ≥ +2: aOR, 5.16; 95% CI, 2.46–10.85) remained associated with mortality. RASS and GCS had similar discrimination for mortality (AUC, 0.683 vs. 0.679; *p* = 0.72), whereas their combination improved discrimination (AUC, 0.712; both comparisons *p* < 0.001). Among patients with GCS 15, mortality was 9.9% with abnormal RASS versus 1.9% with RASS 0. *Conclusions:* RASS demonstrated a bidirectional association with adverse outcomes and provided prognostic information comparable to and complementary to GCS. Its rapid assessment may help identify high-risk older ED patients, including those with GCS 15.

## 1. Introduction

Older adults account for approximately 15–20% of emergency department (ED) visits, and their share has been projected to reach as much as one-third by 2030 [1,2]. They are also at high risk of adverse clinical outcomes [2,3]. Even after accounting for chief complaint and Emergency Severity Index level, older adults had up to twofold higher odds of hospital admission and a 1.9- to 2.5-fold higher hazard of 30-day mortality than adults aged 40–64 years [4]. This population is difficult to triage and risk-stratify because of atypical presentations, multiple comorbidities, polypharmacy, and reduced physiologic reserve [5,6]. Available screening tools, such as Identification of Seniors at Risk and Triage Risk Screening Tool, and physiological early warning scores, such as the National Early Warning Score 2 (NEWS2), show variable performance in predicting hospital admission, return visits, functional decline, and mortality among older ED patients [7,8]. Therefore, simple indicators that can be integrated into routine clinical assessment and support early risk stratification are needed.

Changes in consciousness and arousal may be early signs of a wide range of acute conditions, including infection, neurologic disease, metabolic disorders, and organ failure, particularly in older adults [9,10]. It has been shown that short-term mortality is higher in patients presenting to the ED with a change in level of consciousness compared to those with other common presenting complaints, and that hospitalization and mortality rates are higher in older patients with acute changes in level of consciousness [10,11].

The Glasgow Coma Scale (GCS) was originally developed for patients with traumatic brain injury but has long been widely used as a standard measure of consciousness in the ED [12]. It assesses eye opening and verbal and motor responses. However, it does not directly capture agitation and may have limited ability to distinguish subtle changes in arousal among patients without marked impairment of consciousness.

The Richmond Agitation-Sedation Scale (RASS) is a 10-point scale ranging from +4 to −5 that was originally developed to monitor the depth of sedation in intensive care units (ICU) and has demonstrated high interrater reliability and construct validity [13,14]. The assessment takes only seconds, making it particularly suitable for the ED, and unlike scales that primarily measure depressed consciousness, RASS captures changes toward both agitation and reduced arousal. In a study of older ED patients, paired RASS assessments by trained research personnel and an emergency physician showed moderate interobserver agreement (κ = 0.63) [15]. Interest in the prognostic value of RASS beyond sedation monitoring has increased in recent years [15,16,17]. However, studies in older ED patients have mostly focused on selected populations with acute altered mental status or suspected delirium and on long-term outcomes.

Indirect evidence suggests that RASS may help address the limited ability of GCS to capture agitation and subtle changes in arousal [17]. However, these findings were based on repeated ward observations, the performance of RASS for short-term clinical outcomes and the additional prognostic value it may provide beyond GCS have not been adequately established in unselected older nontraumatic ED patients.

To reduce clinical heterogeneity, the present study focused on nontraumatic presentations, as traumatic conditions, particularly traumatic brain injury in older adults, have distinct clinical presentations, assessment considerations, and prognostic determinants [18]. The primary aim of this study was to evaluate the association of initial RASS values with in-hospital mortality and their prognostic performance in nontraumatic ED patients aged 65 years or older. The secondary aims were to assess the performance of RASS for hospital and intensive care unit admission, compare its performance with GCS, and determine whether RASS provided additional prognostic information beyond GCS.

## 2. Materials and Methods

### 2.1. Study Design and Patients

This was a retrospective, single-center, observational cohort study conducted in the adult ED of a tertiary university hospital with approximately 50,000 ED visits annually. The study was approved by the Gazi University Ethics Committee (approval no. 2025-1955). This study was reported in accordance with the STROBE statement. No study protocol was prospectively registered or publicly published. However, the study design, eligibility criteria, outcomes, and variables were specified in the protocol submitted to and approved by the institutional ethics committee before data extraction and analysis.

The inclusion criteria were age 65 years or older and presentation to the ED for nontraumatic reasons between 1 January 2024, and 31 October 2025. The exclusion criteria applied to the index visit were cardiac arrest before hospital arrival or at ED presentation, missing initial RASS or GCS values, and unavailable clinical outcome data. For patients with more than one ED visit during the study period, only the first visit was designated as the index visit, and each patient was included in the analysis only once. No separate sample size calculation was performed because all eligible patients during the study period were included.

### 2.2. Data Collection and Outcomes

Data were retrospectively obtained from electronic medical records. Age, sex, presenting complaint, RASS and GCS values at presentation, systolic blood pressure, mean arterial pressure [(systolic + 2 × diastolic)/3], heart rate, and peripheral oxygen saturation were recorded. If more than one measurement was available for a variable, the first value recorded at ED presentation was used. Presenting complaints were grouped into clinical categories.

The RASS and GCS scores were recorded by the patient’s attending emergency physician during the routine clinical evaluation upon admission to the ED. The analysis included the first scores recorded before the administration of any medication and reflecting the patient’s neurologic status at presentation. Follow-up scores recorded during observation were not used. RASS ranges from −5 to +4. Positive RASS scores indicate increasing agitation: +1, restless (anxious, with non-aggressive movements); +2, agitated (frequent non-purposeful movements); +3, very agitated (pulling at or removing tubes or catheters, or showing aggressive behavior); and +4, combative (violent and posing an immediate danger to staff). A score of 0 indicates an alert and calm state. Negative scores indicate progressively reduced arousal: −1, drowsy (sustained eye contact in response to voice for more than 10 s); −2, brief eye contact in response to voice lasting less than 10 s; −3, movement or eye opening in response to voice but without eye contact; −4, no response to voice but movement or eye opening in response to physical stimulation; and −5, unarousable (no response to either voice or physical stimulation) [13]. GCS evaluates three components, eye opening, verbal response, and motor response, and provides a total score ranging from 3 to 15 [12]. The scoring criteria for RASS and GCS are summarized in Supplementary Table S1.

The primary outcome was all-cause in-hospital mortality, defined as death in the ED or during the subsequent hospitalization before discharge, within the episode of care beginning with the index ED visit. This outcome was selected because the study specifically aimed to evaluate the association between RASS at ED presentation and mortality during the index episode of acute care. The secondary outcomes were hospital admission and ICU admission. Hospital admission was defined as admission from the ED to either a hospital ward or the ICU. ICU admission was defined as direct admission from the ED to the ICU.

### 2.3. Statistical Analysis

The distribution of continuous variables was assessed using histograms and Q–Q plots. Continuous variables were presented as mean ± standard deviation or median (interquartile range), as appropriate, and categorical variables as number and percentage. For comparisons between groups, the independent samples *t*-test or the Mann–Whitney U test was used for continuous variables, and the Pearson chi-square test or Fisher’s exact test was used for categorical variables.

In-hospital mortality was analyzed as the primary outcome, while hospital and ICU admission were analyzed as secondary outcomes. RASS was treated as a nominal categorical variable to preserve the direction and magnitude of deviations from 0, with RASS 0 as the reference category. Individual RASS values were reported separately in descriptive and univariable analyses. For multivariable models, extreme categories with small event counts were combined to create six nonreference categories (≤−4, −3, −2, −1, +1, and ≥+2). The total GCS score was similarly divided into five categories (15, 14, 13, 9–12, and 3–8), with 15 as the reference category. Unadjusted odds ratios (ORs) and 95% confidence intervals (CIs) were calculated for each category. Because the association between RASS and the outcomes was U-shaped, a single linear trend test across the full score range was not performed. Cochran–Armitage trend tests were conducted separately for the reduced-arousal (RASS 0 to −5) and agitation (RASS 0 to +3) branches.

The prognostic performance of the clinical thresholds was evaluated by calculating sensitivity, specificity, positive and negative predictive values, and positive (LR+) and negative (LR−) likelihood ratios. The 95% CIs for proportions were calculated using the Wilson method, and those for likelihood ratios using the Simel method.

Discrimination was evaluated by receiver operating characteristic (ROC) analysis using the predicted probabilities obtained from logistic regression models for each scale. Numeric category codes were not entered directly into the ROC analysis. Instead, separate models containing RASS, GCS, or both scales were fitted, and the predicted probabilities were saved. The area under the curve (AUC) was calculated from these probabilities. Paired AUCs were compared using the DeLong test. To account for multiple testing across the nine pairwise AUC comparisons, *p* values were adjusted using Holm’s method.

Nested logistic regression models were constructed for multivariable analyses. The baseline clinical model (M0) included age, sex, heart rate, systolic blood pressure, and oxygen saturation. M1 extended M0 by adding GCS, M2 extended M0 by adding RASS, and M3 extended M0 by adding both GCS and RASS. The variables were predefined based on clinical considerations and entered simultaneously using the enter method. The additional contribution of each scale was assessed using likelihood-ratio tests between nested models. Results were reported as adjusted ORs (aOR) with 95% CI.

Multicollinearity was assessed using variance inflation factors (VIF). The agreement between the normal and abnormal classifications of the two scales was assessed using Cohen’s kappa coefficient, based on the thresholds RASS ≠ 0 and GCS < 15. Model fit was assessed using the Hosmer–Lemeshow test. Because some patients had missing vital signs, all multivariable models were fitted using the same complete-case sample of 9435 patients, which included 320 in-hospital deaths. The fully adjusted model estimated 15 predictor parameters: five clinical covariates, four indicator parameters for the five GCS categories, and six indicator parameters for the seven RASS categories. This corresponded to approximately 21 outcome events per parameter, which was considered adequate for conventional logistic regression [19]. No resampling, synthetic oversampling, undersampling, or class weighting was applied because the analyses aimed to estimate associations and prognostic performance in the observed clinical population rather than to optimize classification in an artificially balanced sample. Outcome imbalance does not itself require correction in logistic regression, and resampling may alter the observed outcome prevalence and impair probability calibration without improving discrimination [20].

All reported *p* values were two-sided, and *p* < 0.05 was considered statistically significant. Statistical analyses were performed using IBM SPSS Statistics, version 27.0 (IBM Corp., Armonk, NY, USA). ROC curve analyses, including pairwise comparisons of AUCs using DeLong’s method, were performed in jamovi (version 2.7.38). The same final dataset was used in both programs.

## 3. Results

### 3.1. Study Population

**Figure 1:**
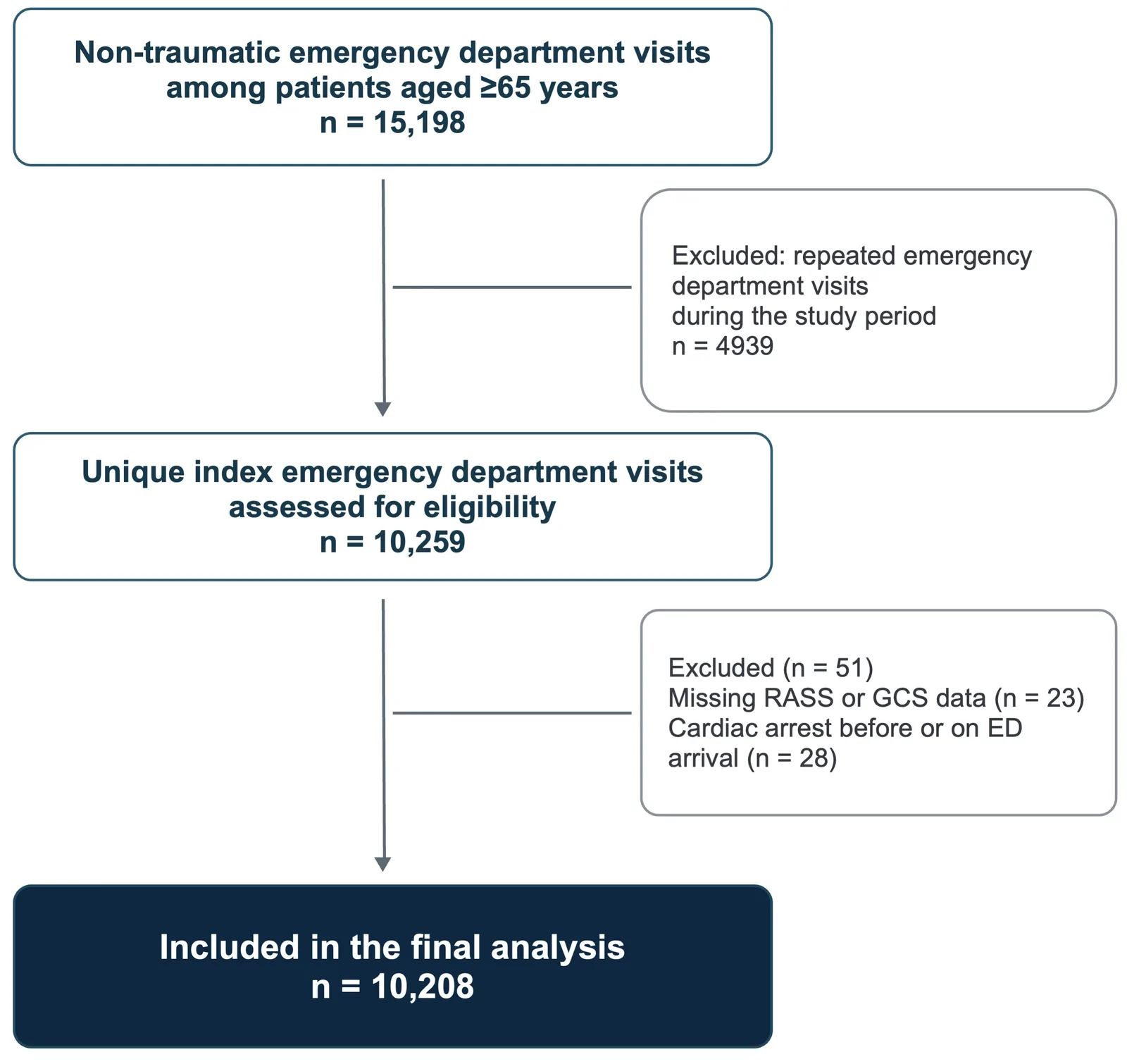
Flow diagram of patient selection and study inclusion.

Of 15,198 nontraumatic ED visits during the study period, 4939 repeat visits and 51 visits meeting the remaining exclusion criteria were excluded, leaving 10,208 unique patients for analysis (Figure 1). The median age was 74 years (interquartile range, 69–81), and 5313 patients (52.0%) were female. In-hospital mortality occurred in 352 patients (3.4%), 2301 (22.5%) were admitted to the hospital, and 856 (8.4%) were admitted to the ICU. Patients who died were older than those who survived, were more often male, and had lower systolic blood pressure and oxygen saturation and higher heart rates at the time of presentation (*p* < 0.001 for all) (Table 1).

### 3.2. Outcomes According to RASS and GCS

**Figure 2:**
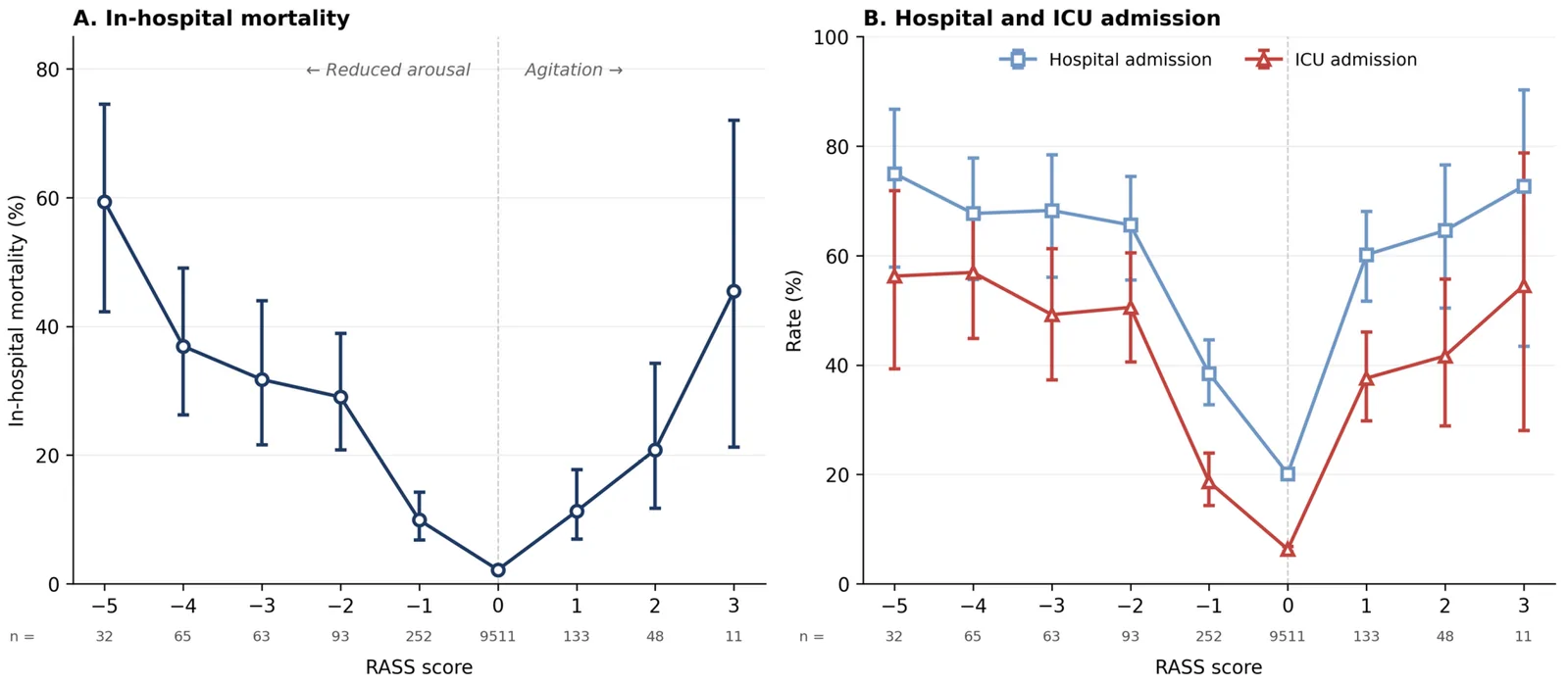
Outcome rates according to RASS score at triage (*n *= 10,208). Error bars represent 95% confidence intervals. The number of patients in each RASS category is shown below the *x*-axis. Cochran–Armitage tests for trend were significant in both the reduced arousal and agitation arms (*p* < 0.001 for all outcomes).

At presentation, RASS was 0 in 9511 patients (93.2%), and GCS was 15 in 9455 patients (92.6%). No patient had a RASS value of +4. Mortality, hospital admission, and ICU admission rates increased in both directions away from RASS 0 (Figure 2). In-hospital mortality was 2.2% among patients with RASS 0 and increased to 59.4% at RASS −5 and 45.5% at RASS +3. Separate trend analyses for the reduced-arousal and agitation branches were significant for all three outcomes (Cochran–Armitage trend test, all *p* < 0.001). Across GCS categories, adverse outcome rates increased as the score decreased; mortality was 2.2% among patients with GCS 15 and 47.3% among those with GCS ≤ 8 (Supplementary Table S2).

**Figure 3:**
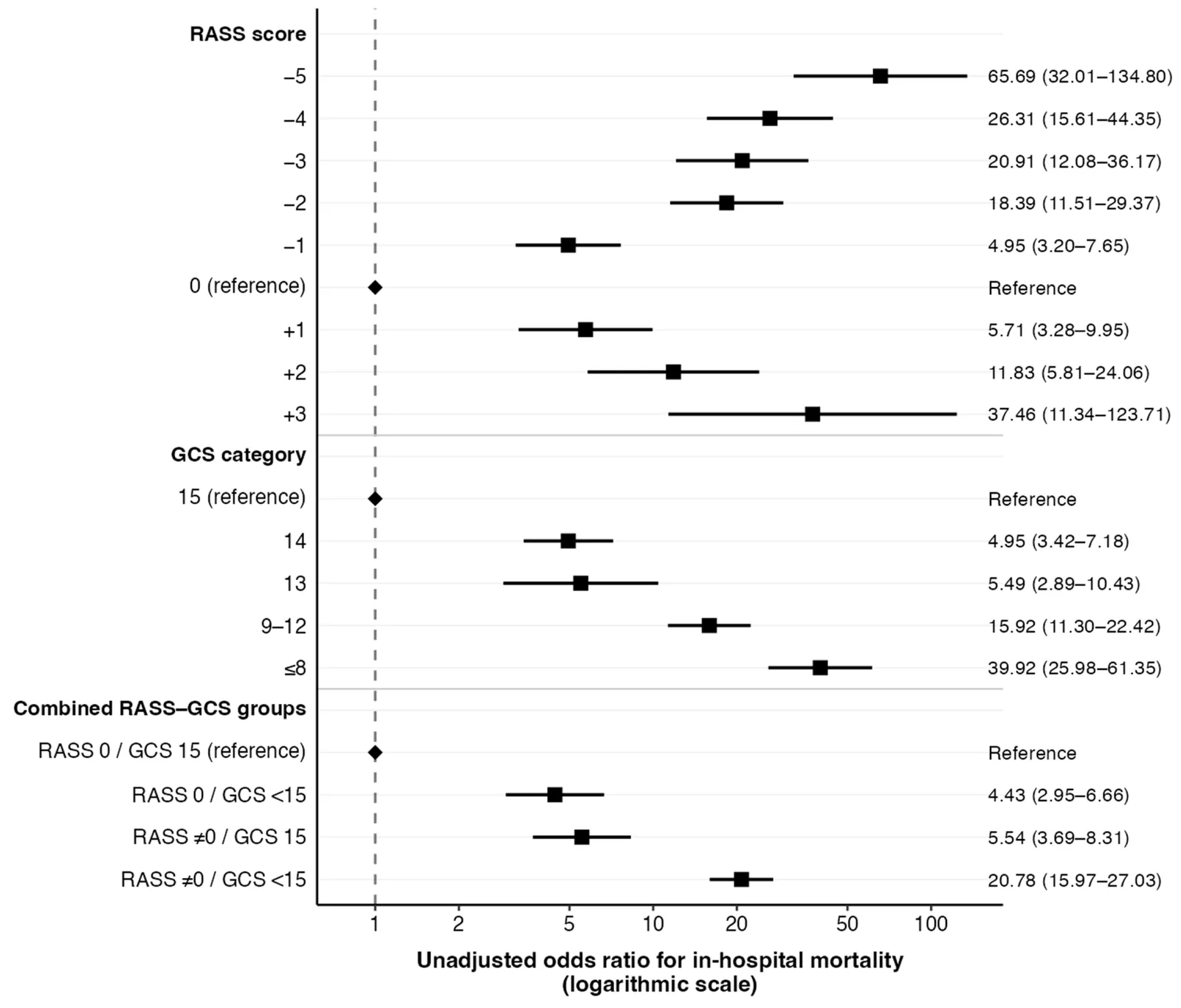
Forest plot of unadjusted odds ratios for in-hospital mortality according to RASS score, GCS category, and combined RASS–GCS groups. Squares indicate odds ratio estimates and horizontal lines indicate 95% confidence intervals; diamonds indicate reference categories. The *x*-axis is presented on a logarithmic scale.

Agreement between abnormal RASS and GCS < 15 was moderate (Cohen’s κ = 0.509). In the four-group analysis, compared with patients with normal values on both scales, the odds of mortality were 4.43 times higher among those with only GCS < 15 (95% CI, 2.95–6.66), 5.54 times higher among those with only abnormal RASS (95% CI, 3.69–8.31), and 20.78 times higher among those with abnormalities on both scales (95% CI, 15.97–27.03). Of the 9455 patients with GCS 15, 303 (3.2%) had an abnormal RASS. Compared with patients with GCS 15 and RASS 0, these patients had higher rates of in-hospital mortality (9.9% vs. 1.9%), ICU admission (25.7% vs. 6.0%), and hospital admission (44.9% vs. 19.7%) (Supplementary Table S2). Unadjusted odds ratios for in-hospital mortality across individual RASS levels, GCS categories, and combined RASS–GCS groups are shown in Figure 3. The corresponding event counts and results for hospital and ICU admission are provided in Supplementary Table S2.

For in-hospital mortality, the RASS ≠ 0 threshold had a sensitivity of 41.2%, a specificity of 94.4%, and an LR+ of 7.36. The corresponding values for the GCS < 15 threshold were 40.9%, 93.8%, and 6.62, respectively (Table 2). Threshold performance for hospital and ICU admission is presented in Supplementary Table S3.

### 3.3. Discriminative Performance of RASS and GCS

RASS and GCS showed similar discrimination for in-hospital mortality (AUC, 0.683 vs. 0.679; *p* = 0.72). The model containing both scales had an AUC of 0.712, which was higher than that of the RASS-only and GCS-only models (*p* < 0.001 for both comparisons). AUC values and pairwise comparisons for all outcomes are presented in Table 3.

### 3.4. Multivariable Analyses

Multivariable models were fitted in a sample of 9435 patients with complete vital sign data. After adjustment for age, sex, heart rate, systolic blood pressure, oxygen saturation, and GCS, both negative and positive RASS categories remained associated with in-hospital mortality. In the combined model, compared with RASS 0, the aORs were 1.81 for RASS −1 (95% CI, 1.07–3.09), 4.93 for RASS ≤ −4 (95% CI, 2.10–11.61), 2.62 for RASS +1 (95% CI, 1.38–4.97), and 5.16 for RASS ≥ +2 (95% CI, 2.46–10.85) (Table 4).

Adding either scale to the baseline clinical model (M0) significantly improved model fit (GCS: likelihood-ratio χ^2^ = 160.9, df = 4; RASS: χ^2^ = 176.9, df = 6; both *p* < 0.001). RASS also provided a significant contribution when added to the model containing GCS (χ^2^ = 38.0, df = 6, *p* < 0.001), and the contribution of GCS to the model containing RASS was also significant (χ^2^ = 22.0, df = 4, *p* < 0.001). All VIF values were below 2.4, and the Hosmer-Lemeshow test indicated adequate fit of the combined model (χ^2^ = 10.12, df = 8, *p* = 0.256).

In the adjusted analyses of hospital and ICU admission, RASS provided additional information beyond GCS, and GCS provided additional information beyond RASS (all *p* < 0.001; Supplementary Tables S4 and S5).

## 4. Discussion

In this study, among ED presentations of patients aged 65 and older, an abnormal RASS value at presentation was significantly associated with in-hospital mortality, hospital admission, and ICU admission. These associations remained significant after adjustment for age, sex, and vital signs. RASS and GCS showed similar discrimination for in-hospital mortality, while the combination of the two scales provided greater discrimination for all outcomes than either scale alone.

A major strength of this study is that RASS was routinely recorded in an unselected cohort of older ED patients rather than in a sample preselected for altered mental status. Our findings are consistent with previous studies showing an association between impaired arousal at presentation and mortality in older patients. However, these studies have mainly focused on selected patients with altered mental status, hospitalized patients, or long-term mortality [10,17,21,22]. Our study demonstrates this association for in-hospital mortality, hospital admission, and ICU admission in a large, unselected population of older nontraumatic ED patients.

By preserving the full direction and magnitude of RASS, our analysis showed a U-shaped relationship, with higher rates of in-hospital mortality, hospital admission, and ICU admission on both the negative and positive sides of RASS 0. In-hospital mortality was 2.2% among patients with RASS 0 and increased to 59.4% at RASS −5 and 45.5% at RASS +3. The same bidirectional pattern was observed for hospital and ICU admission. This finding suggests that not only reduced arousal but also deviations toward agitation may indicate greater acute illness severity in older ED patients. In a substantial proportion of previous studies, impaired arousal was evaluated dichotomously as normal or abnormal RASS, the direction of positive and negative deviations was not preserved, only reduced arousal was considered, or positive RASS values were included in the same category as RASS 0 [10,16,22,23]. Bellelli et al. found that impaired arousal was associated with increased 1- and 6-month mortality among patients admitted to an acute geriatric unit [22]. They used a modified RASS and included deviations in both positive and negative directions in the abnormal arousal group. In a study of 1084 older ED patients, Han et al. found that impaired arousal assessed by RASS at presentation was independently associated with 6-month mortality, and this association remained present among patients without delirium [16]. Zadravecz et al. conducted one of the few studies that evaluated the positive and negative RASS branches separately. In hospitalized ward patients, RASS values above and below 0 were entered separately into regression models, and both were associated with 24-h mortality [17]. This bidirectional pattern suggests that modeling RASS as a single linear variable may underestimate its prognostic value by obscuring the risk associated with positive scores.

Some studies have reported that RASS provides prognostic information similar to GCS [17,23,24,25]. In a study of 4120 mechanically ventilated patients, Rakhit et al. showed that a SOFA score using RASS for its neurologic component had similar discrimination for mortality to the GCS-based SOFA score [24]. However, these data were obtained from a selected ICU population. In a large study of general ward patients, RASS and GCS showed similar discrimination for in-hospital mortality, while their combined use improved discrimination [17]. Our findings extend these observations directly to an older ED population. RASS and GCS showed similar discrimination for in-hospital mortality (AUC, 0.683 vs. 0.679; *p* = 0.72), whereas the combined model provided better discrimination than either scale alone (AUC, 0.712; *p* < 0.001 for both comparisons). The combined use of the two scales also provided greater discrimination for hospital and ICU admission, although their overall performance for these outcomes was low. In models adjusted for age, sex, and vital signs, each scale also provided significant information beyond the other (RASS added to GCS: likelihood-ratio χ^2^ = 38.0, df = 6, *p* < 0.001; GCS added to RASS: χ^2^ = 22.0, df = 4, *p* < 0.001). Therefore, RASS should be interpreted as a brief neurologic assessment that complements current clinical evaluation rather than as a stand-alone prognostic tool that replaces clinical variables or GCS.

The subgroup analyses further showed that RASS and GCS are not fully interchangeable. Among patients with normal values on both scales, mortality was 1.9%, hospital admission was 19.7%, and ICU admission was 6.0%. In contrast, among the 303 patients with an abnormal RASS but a GCS of 15, these rates were 9.9%, 44.9%, and 25.7%, respectively. Adverse outcomes were also more frequent among patients with RASS 0 and GCS < 15. Much of this disagreement may be explained by the ability of RASS to assess restlessness and agitation, which are not directly captured by GCS. In addition, mild changes in arousal, fluctuations, and behavioral abnormalities may not be reflected in the total GCS score in routine clinical practice [12,26].

Threshold analyses provide additional information on how RASS may be used clinically. Category-specific adjusted odds ratios represent relative associations rather than patient-specific probabilities or clinical decision thresholds, and the wide confidence intervals at the extreme RASS levels limit the precision of the estimated magnitude. For in-hospital mortality, the RASS ≠ 0 threshold had a sensitivity of 41.2%, a specificity of 94.4%, and a positive likelihood ratio of 7.36. These findings suggest that any deviation from RASS 0 may serve as a practical initial warning sign. However, because of its low sensitivity and limited negative likelihood ratio, a normal RASS cannot be used to rule out an adverse outcome. Therefore, RASS should be interpreted as a warning sign that prompts further assessment, rather than as a stand-alone rule-out test or a definitive clinical decision threshold. Given that acute mental status disorders and delirium are common in older ED patients, often go unrecognized, and are associated with adverse outcomes even after discharge [27,28,29,30], a scale with a positive likelihood ratio above 7 that is also quick and simple to administer has practical value, particularly in patients who might be considered low risk because their GCS is normal. Additionally, the simple structure of RASS and its discrimination comparable to GCS suggest that it could serve as the bedside assessment of arousal in older ED patients.

Physiological early warning scores such as NEWS2 quantify derangement across multiple vital-sign domains and incorporate a categorical assessment of consciousness or new confusion. The applicability of NEWS2 to older adults, particularly those with frailty and multimorbidity, remains an area of ongoing evaluation [31]. In a multicenter ED study, the discrimination of NEWS2 for in-hospital mortality was significantly lower in patients aged 65 years or older than in adults aged 18–64 years (AUROC, 0.778 vs. 0.842; *p* < 0.001) [32]. NEWS2 has also been evaluated for outcomes similar to those examined in the present study. In a multicenter study of 932 nontraumatic ED patients aged 65 years or older, NEWS2 yielded AUCs of 0.679 for hospital admission, 0.693 for ICU admission, and 0.726 for in-hospital mortality [33]. In a prospective study restricted to frail ED patients aged 75 years or older, NEWS2 yielded AUCs of 0.62 for hospital admission, 0.72 for high-dependency unit admission, and 0.70 for 30-day mortality [34]. For descriptive context, the RASS AUCs in our cohort were 0.566 for hospital admission, 0.628 for ICU admission, and 0.683 for in-hospital mortality. The reported NEWS2 estimates were therefore numerically higher, particularly for admission outcomes. However, these estimates were derived from different cohorts and, in the latter study, partly different outcome definitions; therefore, they should not be interpreted as a direct head-to-head comparison or as evidence of comparative superiority.

RASS differs from NEWS2 in both scope and purpose. Whereas NEWS2 provides a composite assessment of physiological derangement, RASS provides a focused assessment of a single clinical domain: the level of arousal. Accordingly, the numerical comparisons above should be interpreted as clinical context rather than as comparisons between interchangeable instruments. Therefore, the present findings should not be interpreted as establishing RASS as an alternative to NEWS2 or other multidomain risk-stratification instruments. Rather, the association of RASS with clinical outcomes after adjustment for age, sex, vital signs, and GCS suggests that altered arousal may provide complementary prognostic information during the initial ED assessment.

### Limitations

This study has several limitations. First, its retrospective and single-center design may limit the generalizability of the findings to other ED settings. Because patients presenting with trauma were excluded, these findings are limited to older adults with nontraumatic ED presentations and should not be extrapolated to patients with traumatic injuries, particularly traumatic brain injury. Excluding patients without recorded RASS or GCS values may have introduced selection bias. Because of missing vital sign data, multivariable analyses were conducted in a sample of 9435 patients, and multiple imputation was not performed. Potential confounders such as comorbidity burden, frailty, dementia, baseline functional and cognitive status, and the use of psychoactive medications or sedatives could not be reliably assessed. Because RASS and GCS values were obtained from routine clinical records, physician training, measurement conditions and interrater reliability could not be verified. Only measurements obtained at presentation were included, and the prognostic value of serial changes in RASS and GCS was not evaluated. Other structured assessments of consciousness, such as the FOUR score [35], were not recorded in our cohort and merit evaluation in future studies. The small number of patients with markedly positive RASS values and the absence of patients with RASS +4 reduced the precision of estimates on the agitation side and resulted in wide confidence intervals for some categories. In-hospital mortality is influenced by the duration of hospitalization and local discharge practices and does not capture deaths occurring after discharge; therefore, our findings should not be extrapolated to mortality within a fixed follow-up period, such as 30 days. Finally, hospital and ICU admission are influenced not only by clinical illness severity but also by local decision-making processes and resource availability.

## 5. Conclusions

Among older patients presenting to the ED for nontraumatic reasons, an abnormal RASS value at presentation was associated with increased in-hospital mortality, hospital admission, and ICU admission. RASS and GCS showed similar discrimination, while their combined use provided additional prognostic information. Therefore, RASS may be considered a brief measure for the rapid initial assessment of arousal and may provide complementary information alongside GCS when a more comprehensive assessment is needed.

## Figures and Tables

**Table 1:** Baseline characteristics of the study population according to in-hospital mortality.

Characteristic	All Patients (*n* = 10,208)	Survivors (*n* = 9856)	In-Hospital Mortality (*n* = 352)	*p* Value
Age, years, median (IQR)	74 (69–81)	74 (69–80)	79 (73–86)	<0.001
Age ≥ 85 years, *n* (%)	1451 (14.2)	1340 (13.6)	111 (31.5)	<0.001
Female sex, *n* (%)	5313 (52.0)	5167 (52.4)	146 (41.5)	<0.001
Vital signs on arrival, median (IQR) *				
Systolic blood pressure, mmHg	138 (121–158)	139 (122–158)	120 (100–141)	<0.001
Mean arterial pressure	98.3 (88.0–109.0)	98.7 (88.3–109.0)	88.0 (76.0–102.9)	<0.001
Heart rate, beats/min	86 (74–98)	86 (74–98)	100 (87–116)	<0.001
Oxygen saturation, %	96 (94–97)	96 (94–97)	93 (89–96)	<0.001
Presenting complaint category, *n* (%)				<0.001
Respiratory	2179 (21.3)	2042 (20.7)	137 (38.9)	
Gastrointestinal	2733 (26.8)	2671 (27.1)	62 (17.6)	
Cardiovascular	1214 (11.9)	1191 (12.1)	23 (6.5)	
Neurological	1192 (11.7)	1161 (11.8)	31 (8.8)	
Non-specific/general	1139 (11.2)	1060 (10.8)	79 (22.4)	
Genitourinary	519 (5.1)	515 (5.2)	4 (1.1)	
Musculoskeletal	330 (3.2)	328 (3.3)	2 (0.6)	
Head and neck	324 (3.2)	323 (3.3)	1 (0.3)	
Other **	578 (5.7)	565 (5.7)	13 (3.7)	
Richmond Agitation-Sedation Scale				
RASS = 0 (normal)	9511 (93.2)	9304 (94.4)	207 (58.8)	<0.001
RASS ≠ 0 (any abnormal)	697 (6.8)	552 (5.6)	145 (41.2)	
Negative RASS (−1 to −5)	505 (4.9)	390 (4.0)	115 (32.7)	<0.001
Positive RASS (+1 to +3)	192 (1.9)	162 (1.6)	30 (8.5)	<0.001
Glasgow Coma Scale				
Total score, median (IQR)	15 (15–15)	15 (15–15)	15 (12–15)	<0.001
GCS < 15, *n* (%)	753 (7.4)	609 (6.2)	144 (40.9)	<0.001
GCS ≤ 13, *n* (%)	394 (3.9)	286 (2.9)	108 (30.7)	<0.001
GCS ≤ 8, *n* (%)	93 (0.9)	49 (0.5)	44 (12.5)	<0.001
Outcomes, *n* (%)				
Hospital admission	2301 (22.5)	1979 (20.1)	322 (91.5)	<0.001
Intensive care unit admission	856 (8.4)	642 (6.5)	214 (60.8)	<0.001
Death in the emergency department	30 (0.3)	0 (0.0)	30 (8.5)	

IQR: interquartile range; RASS: Richmond Agitation–Sedation Scale; GCS: Glasgow Coma Scale. Continuous variables were compared with the Mann–Whitney U test and categorical variables with the chi-square test. * Vital signs were available in 9497–9535 patients. ** Other includes dermatological/allergic (*n* = 161), metabolic/endocrine (*n* = 71), psychiatric (*n* = 51), and unclassifiable (*n* = 295) complaints.

**Table 2:** Prognostic performance of clinical thresholds of RASS and GCS for in-hospital mortality.

Threshold	Sensitivity% (95% CI)	Specificity% (95% CI)	PPV% (95% CI)	NPV% (95% CI)	LR+ (95% CI)	LR− (95% CI)
RASS ≠ 0	41.2 (36.0–46.5)	94.4 (93.9–94.8)	20.8 (17.8–24.0)	97.8 (97.5–98.1)	7.36 (6.34–8.54)	0.62 (0.57–0.68)
Negative RASS (<0)	32.7 (28.0–37.7)	96.0 (95.6–96.4)	22.8 (19.3–26.6)	97.6 (97.2–97.8)	8.26 (6.90–9.87)	0.70 (0.65–0.75)
Positive RASS (>0)	8.5 (6.0–11.9)	98.4 (98.1–98.6)	15.6 (11.2–21.4)	96.8 (96.4–97.1)	5.19 (3.56–7.54)	0.93 (0.90–0.96)
GCS < 15	40.9 (35.7–46.2)	93.8 (93.3–94.3)	19.1 (16.5–22.1)	97.8 (97.5–98.1)	6.62 (5.71–7.67)	0.63 (0.58–0.69)
GCS ≤ 13	30.7 (26.1–35.7)	97.1 (96.7–97.4)	27.4 (23.2–32.0)	97.5 (97.2–97.8)	10.57 (8.71–12.84)	0.71 (0.67–0.77)
GCS ≤ 8	12.5 (9.4–16.4)	99.5 (99.3–99.6)	47.3 (37.5–57.4)	97.0 (96.6–97.3)	25.14 (16.97–37.25)	0.88 (0.85–0.91)

PPV: positive predictive value; NPV: negative predictive value; LR+: positive likelihood ratio; LR−: negative likelihood ratio; CI: confidence interval; RASS: Richmond Agitation-Sedation Scale; GCS: Glasgow Coma Scale. Proportions are shown with Wilson 95% confidence intervals; likelihood ratios with Simel 95% confidence intervals.

**Table 3:** Discrimination of RASS, GCS, and combined models for each outcome.

Outcome	Model	AUC (95% CI)	Comparison	DeLong *p* Value	Holm *p* Value
In-hospital mortality	RASS	0.683 (0.656–0.709)	RASS vs. GCS	0.719	0.719
	GCS	0.679 (0.652–0.705)	RASS+GCS vs. GCS	<0.001	<0.001
	RASS+GCS	0.712 (0.689–0.740)	RASS+GCS vs. RASS	<0.001	<0.001
Hospital admission	RASS	0.566 (0.558–0.574)	RASS vs. GCS	0.004	0.008
	GCS	0.555 (0.547–0.563)	RASS+GCS vs. GCS	<0.001	<0.001
	RASS+GCS	0.576 (0.567–0.585)	RASS+GCS vs. RASS	<0.001	<0.001
ICU admission	RASS	0.628 (0.613–0.644)	RASS vs. GCS	0.002	0.007
	GCS	0.608 (0.593–0.623)	RASS+GCS vs. GCS	<0.001	<0.001
	RASS+GCS	0.645 (0.628–0.662)	RASS+GCS vs. RASS	<0.001	<0.001

AUC: Area under the curve; CI: Confidence interval; RASS: Richmond Agitation-Sedation Scale; GCS: Glasgow Coma Scale. Each scale was entered as a nominal categorical variable (reference: RASS 0; GCS 15); ROC analysis was performed on the predicted probabilities of these logistic models. AUCs were compared with the DeLong test for paired ROC curves. *p* values were adjusted across the nine comparisons using Holm’s method.

**Table 4:** Multivariable logistic regression models for in-hospital mortality.

Variable	M1: Clinical + GCS, aOR (95% CI)	M2: Clinical + RASS, aOR (95% CI)	M3: Clinical + GCS + RASS, aOR (95% CI)
Age, per year	1.04 (1.03–1.06)	1.05 (1.03–1.06)	1.04 (1.03–1.06)
Male sex	1.63 (1.27–2.10)	1.60 (1.24–2.06)	1.63 (1.27–2.10)
Heart rate, per 10 beats/min	1.24 (1.18–1.30)	1.24 (1.18–1.30)	1.23 (1.17–1.29)
Systolic blood pressure, per 10 mmHg	0.82 (0.78–0.85)	0.81 (0.78–0.85)	0.82 (0.78–0.86)
Oxygen saturation, per %	0.93 (0.92–0.95)	0.94 (0.92–0.95)	0.94 (0.92–0.95)
GCS category			
15	Reference	—	Reference
14	3.52 (2.34–5.31)	—	2.52 (1.60–3.97)
13	3.50 (1.75–7.01)	—	1.68 (0.77–3.66)
9–12	7.84 (5.24–11.72)	—	2.92 (1.65–5.16)
3–8	12.90 (7.52–22.11)	—	3.60 (1.51–8.58)
RASS category			
0	—	Reference	Reference
−1	—	2.69 (1.65–4.38)	1.81 (1.07–3.09)
−2	—	9.77 (5.74–16.64)	4.58 (2.37–8.85)
−3	—	7.60 (4.02–14.35)	2.95 (1.31–6.62)
≤−4	—	14.57 (8.50–24.95)	4.93 (2.10–11.61)
+1	—	2.73 (1.44–5.20)	2.62 (1.38–4.97)
≥+2	—	9.18 (4.68–18.03)	5.16 (2.46–10.85)
AUC (95% CI)	0.855 (0.833–0.876)	0.853 (0.831–0.876)	0.858 (0.836–0.880)

aOR: adjusted odds ratio; CI: confidence interval; RASS: Richmond Agitation-Sedation Scale; GCS: Glasgow Coma Scale. The clinical model comprised age, sex, heart rate, systolic blood pressure, and oxygen saturation. All models were fitted on the same complete-case sample (*n* = 9435), which included 320 in-hospital deaths, to ensure comparability. Adding RASS to the clinical model improved model fit (likelihood-ratio χ^2^ = 176.9, df = 6, *p* < 0.001), as did adding GCS (χ^2^ = 160.9, df = 4, *p* < 0.001). RASS provided additional information beyond GCS (χ^2^ = 38.0, df = 6, *p* < 0.001) and GCS beyond RASS (χ^2^ = 22.0, df = 4, *p* < 0.001).

## Data Availability

The data are not publicly available because of institutional and patient-privacy restrictions. Deidentified data may be available from the corresponding author upon reasonable request and subject to institutional approval.

## Supplementary Materials

The following supporting information can be downloaded at: https://www.mdpi.com/article/10.3390/medicina62091803/s1. Supplementary Table S1: Scoring criteria for the Richmond Agitation–Sedation Scale and Glasgow Coma Scale; Supplementary Table S2: Clinical outcomes according to RASS score, GCS category, and combined RASS–GCS groups; Supplementary Table S3: Prognostic performance of clinical thresholds of RASS and GCS for hospital admission and ICU admission; Supplementary Table S4: Multivariable logistic regression models for hospital admission; Supplementary Table S5: Multivariable logistic regression models for intensive care unit admission.

## Abbreviations

The following abbreviations are used in this manuscript:

ED	Emergency department
ICU	Intensive care unit
RASS	Richmond Agitation-Sedation Scale
GCS	Glasgow Coma Scale
ROC	Receiver operating characteristic
AUC	Area under the curve
LR	Likelihood ratio
OR	Odds ratio
CI	Confidence interval
PPV	Positive predictive value
NPV	Negative predictive value
NEWS2	National Early Warning Score 2
