# Supplementary material for: Prognostic Value of the Richmond Agitation-Sedation Scale for Mortality and Admission in Older Emergency Department Patients
Source: Medicina (Kaunas). 2026 Sep 19;62(9):1803. doi: 10.3390/medicina62091803 (PMC13608948; doi:10.3390/medicina62091803)
Supplement: Supplementary file 1 [file medicina-62-01803-s001.zip › medicina-4542993-supplementary.pdf]

Supplementary Table S1. Scoring criteria for the Richmond Agitation–Sedation Scale and Glasgow Coma Scale

**RASS score**	**Description**
**+4**	Combative: Violent and posing an immediate danger to staff
**+3**	Very agitated: Pulling at or removing tubes or catheters, or showing aggressive behavior
**+2**	Agitated: Frequent non-purposeful movements
**+1**	Restless: Anxious, with non-aggressive movements
**0**	Alert and calm
**−1**	Drowsy: Sustained eye contact in response to voice for more than 10 seconds
**−2**	Brief eye contact in response to voice lasting less than 10 seconds
**−3**	Movement or eye opening in response to voice but without eye contact
**−4**	No response to voice, but movement or eye opening in response to physical stimulation
**−5**	Unarousable: No response to either voice or physical stimulation

**Component**	**Score**	**Description**
**Eye response**	4	Eyes open without stimulation
	3	Eye opening occurs in response to a spoken or auditory stimulus
	2	Eye opening occurs only after the application of physical pressure
	1	No eye opening
**Verbal response**	5	Responds appropriately and is oriented to person, place, and time
	4	Communicates verbally but is confused or disoriented
	3	Produces recognizable words without coherent conversation
	2	Produces vocal sounds without recognizable words
	1	No verbal response
**Motor response**	6	Performs a requested movement

	5	Purposefully moves toward the site of applied pressure
	4	Withdraws from pressure with normal flexion
	3	Shows abnormal flexion in response to pressure
	2	Shows extension in response to pressure
	1	No motor response
**Total**	3-15	

Supplementary Table S2. Clinical outcomes according to RASS score, GCS category, and combined RASS-GCS groups.

	Total N (%)	In-hospital mortality		Hospital admission		ICU admission	
		N (%)	OR (95% CI)	N (%)	OR (95% CI)	N (%)	OR (95% CI)
**RASS score**							
−5	32 (0.3)	19 (59.4)	65.69 (32.01-134.80)	24 (75.0)	11.92 (5.34-26.56)	18 (56.2)	19.09 (9.45-38.58)
−4	65 (0.6)	24 (36.9)	26.31 (15.61-44.35)	44 (67.7)	8.32 (4.94-14.03)	37 (56.9)	19.63 (11.93-32.29)
−3	63 (0.6)	20 (31.7)	20.91 (12.08-36.17)	43 (68.3)	8.54 (5.01-14.55)	31 (49.2)	14.39 (8.72-23.74)
−2	93 (0.9)	27 (29.0)	18.39 (11.51-29.37)	61 (65.6)	7.57 (4.92-11.65)	47 (50.5)	15.17 (10.02-22.98)
−1	252 (2.5)	25 (9.9)	4.95 (3.20-7.65)	97 (38.5)	2.49 (1.92-3.22)	47 (18.7)	3.41 (2.45-4.72)
0	9,511 (93.2)	207 (2.2)	Reference	1,913 (20.1)	Reference	600 (6.3)	Reference
+1	133 (1.3)	15 (11.3)	5.71 (3.28-9.95)	80 (60.2)	6.00 (4.22-8.51)	50 (37.6)	8.95 (6.24-12.83)
+2	48 (0.5)	10 (20.8)	11.83 (5.81-24.06)	31 (64.6)	7.24 (4.00-13.11)	20 (41.7)	10.61 (5.94-18.94)
+3	11 (0.1)	5 (45.5)	37.46 (11.34-123.71)	8 (72.7)	10.59 (2.81-39.96)	6 (54.5)	17.82 (5.42-58.57)
**GCS category**							
15	9,455 (92.6)	208 (2.2)	Reference	1,938 (20.5)	Reference	628 (6.6)	Reference
14	359 (3.5)	36 (10.0)	4.95 (3.42-7.18)	129 (35.9)	2.18 (1.74-2.71)	58 (16.2)	2.71 (2.02-3.63)
13	100 (1.0)	11 (11.0)	5.49 (2.89-10.43)	42 (42.0)	2.81 (1.88-4.19)	24 (24.0)	4.44 (2.79-7.07)
9-12	201 (2.0)	53 (26.4)	15.92 (11.30-22.42)	126 (62.7)	6.52 (4.88-8.71)	90 (44.8)	11.40 (8.53-15.22)
≤ 8	93 (0.9)	44 (47.3)	39.92 (25.98-61.35)	66 (71.0)	9.48 (6.04-14.88)	56 (60.2)	21.27 (13.94-32.48)
**Combined groups**							
RASS=0 and GCS=15	9152 (89.7)	178 (1.9)	Reference	1,802 (19.7)	Reference	550 (6.0)	Reference
RASS=0 and GCS<15	359 (3.5)	29 (8.1)	4.43 (2.95-6.66)	111 (30.9)	1.83 (1.45-2.30)	50 (13.9)	2.53 (1.85-3.45)
RASS≠0 and GCS=15	303 (3.0)	30 (9.9)	5.54 (3.69-8.31)	136 (44.9)	3.32 (2.63-4.19)	78 (25.7)	5.42 (4.13-7.11)
RASS≠0 and GCS<15	394 (3.9)	115 (29.2)	20.78 (15.97-27.03)	252 (64.0)	7.24 (5.86-8.95)	178 (45.2)	12.89 (10.38-16.00)

OR: odds ratio; CI: confidence interval; RASS: Richmond Agitation–Sedation Scale; GCS: Glasgow Coma Scale. Percentages in the "Total" column are of the entire cohort (n = 10,208); percentages in the outcome columns are within-category (row) percentages. Unadjusted odds ratios with the specified reference category. Global chi-square p<0.001 for all three panels and all three outcomes.

Supplementary Table S3. Prognostic performance of clinical thresholds of RASS and GCS for hospital admission and ICU admission

Outcome	Threshold	Sensitivity % (95% CI)	Specificity % (95% CI)	PPV % (95% CI)	NPV % (95% CI)	LR+ (95% CI)	LR− (95% CI)
Hospital admission	RASS ≠ 0	16.9 (15.4-18.4)	96.1 (95.6-96.5)	55.7 (52.0-59.3)	79.9 (79.1-80.7)	4.31 (3.74-4.97)	0.87 (0.85-0.88)
	Negative RASS (<0)	11.7 (10.4-13.1)	97.0 (96.6-97.4)	53.3 (48.9-57.6)	79.1 (78.2-79.9)	3.92 (3.31-4.64)	0.91 (0.90-0.92)
	Positive RASS (>0)	5.2 (4.3-6.2)	99.1 (98.8-99.3)	62.0 (54.9-68.5)	78.2 (77.4-79.0)	5.60 (4.20-7.47)	0.96 (0.95-0.97)
	|RASS| ≥ 2	9.2 (8.1-10.4)	98.7 (98.5-98.9)	67.6 (62.2-72.6)	78.9 (78.1-79.7)	7.18 (5.69-9.06)	0.92 (0.91-0.93)
	GCS < 15	15.8 (14.3-17.3)	95.1 (94.6-95.5)	48.2 (44.7-51.8)	79.5 (78.7-80.3)	3.20 (2.79-3.66)	0.89 (0.87-0.90)
	GCS ≤ 13	10.2 (9.0-11.5)	98.0 (97.6-98.3)	59.4 (54.5-64.1)	78.9 (78.1-79.7)	5.03 (4.13-6.11)	0.92 (0.90-0.93)
	GCS ≤ 8	2.9 (2.3-3.6)	99.7 (99.5-99.8)	71.0 (61.1-79.2)	77.9 (77.1-78.7)	8.40 (5.38-13.11)	0.97 (0.97-0.98)
ICU admission	RASS ≠ 0	29.9 (26.9-33.1)	95.3 (94.8-95.7)	36.7 (33.2-40.4)	93.7 (93.2-94.2)	6.34 (5.53-7.27)	0.74 (0.70-0.77)
	Negative RASS (<0)	21.0 (18.4-23.9)	96.5 (96.1-96.9)	35.6 (31.6-39.9)	93.0 (92.5-93.5)	6.05 (5.11-7.16)	0.82 (0.79-0.85)
	Positive RASS (>0)	8.9 (7.2-11.0)	98.8 (98.5-99.0)	39.6 (32.9-46.6)	92.2 (91.7-92.7)	7.16 (5.41-9.48)	0.92 (0.90-0.94)
	|RASS| ≥ 2	18.6 (16.1-21.3)	98.4 (98.1-98.6)	51.0 (45.4-56.5)	93.0 (92.4-93.4)	11.35 (9.20-14.02)	0.83 (0.80-0.85)
	GCS < 15	26.6 (23.8-29.7)	94.4 (93.9-94.8)	30.3 (27.1-33.7)	93.4 (92.8-93.8)	4.74 (4.13-5.45)	0.78 (0.75-0.81)
	GCS ≤ 13	19.9 (17.3-22.7)	97.6 (97.3-97.9)	43.1 (38.3-48.1)	93.0 (92.5-93.5)	8.29 (6.88-9.99)	0.82 (0.79-0.85)
	GCS ≤ 8	6.5 (5.1-8.4)	99.6 (99.5-99.7)	60.2 (50.1-69.6)	92.1 (91.5-92.6)	16.54 (10.98-24.90)	0.94 (0.92-0.96)

PPV: positive predictive value; NPV: negative predictive value; LR+: positive likelihood ratio; LR−: negative likelihood ratio; ICU: intensive care unit; RASS: Richmond Agitation–Sedation Scale; GCS: Glasgow Coma Scale.
Proportions are shown with Wilson 95% confidence intervals; likelihood ratios with Simel 95% confidence intervals.

Supplementary Table S4. Multivariable logistic regression models for hospital admission

Variable	M1: Clinical + GCS aOR (95% CI)	M2: Clinical + RASS aOR (95% CI)	M3: Clinical + GCS + RASS aOR (95% CI)
Age, per year	1.02 (1.01-1.03)	1.02 (1.02-1.03)	1.02 (1.01-1.03)
Male sex	1.45 (1.31-1.61)	1.46 (1.31-1.61)	1.46 (1.32-1.62)
Heart rate, per 10 beats/min	1.13 (1.10-1.15)	1.12 (1.09-1.15)	1.12 (1.09-1.15)
Systolic blood pressure, per 10 mmHg	0.95 (0.93-0.97)	0.95 (0.93-0.96)	0.95 (0.93-0.96)
Oxygen saturation, per %	0.91 (0.90-0.92)	0.92 (0.91-0.93)	0.92 (0.91-0.93)
**GCS category:**			
15	Reference	—	Reference
14	1.78 (1.40-2.26)	—	1.52 (1.18-1.96)
13	1.92 (1.25-2.95)	—	1.22 (0.76-1.95)
9–12	4.36 (3.15-6.02)	—	2.20 (1.47-3.31)
3–8	5.09 (3.09-8.37)	—	1.80 (0.86-3.77)
**RASS category:**			
0	—	Reference	Reference
−1	—	1.72 (1.30-2.29)	1.45 (1.07-1.95)
−2	—	4.62 (2.91-7.33)	2.92 (1.74-4.92)
−3	—	4.61 (2.60-8.17)	2.59 (1.33-5.07)
≤−4	—	6.14 (3.76-10.03)	3.49 (1.70-7.17)
+1	—	3.79 (2.56-5.61)	3.70 (2.49-5.48)
≥+2	—	5.19 (2.89-9.32)	3.74 (2.02-6.92)
AUC (95% CI)	0.692 (0.678-0.705)	0.694 (0.681-0.707)	0.695 (0.682-0.709)

aOR, adjusted odds ratio; RASS: Richmond Agitation–Sedation Scale; GCS: Glasgow Coma Scale. The clinical model comprised age, sex, heart rate, systolic blood pressure, and oxygen saturation.

Likelihood-ratio tests: adding RASS to the clinical model $\chi^2=200.0$, df=6, $p<0.001$; adding GCS $\chi^2=143.5$, df=4, $p<0.001$; RASS beyond GCS $\chi^2=78.2$, df=6, $p<0.001$; GCS beyond RASS $\chi^2=21.7$, df=4, $p<0.001$.

Supplementary Table S5. Multivariable logistic regression models for intensive care unit admission

Variable	M1: Clinical + GCS aOR (95% CI)	M2: Clinical + RASS aOR (95% CI)	M3: Clinical + GCS + RASS aOR (95% CI)
Age, per year	1.03 (1.02-1.04)	1.03 (1.02-1.04)	1.03 (1.02-1.04)
Male sex	1.48 (1.26-1.73)	1.47 (1.25-1.73)	1.48 (1.26-1.74)
Heart rate, per 10 beats/min	1.17 (1.13-1.21)	1.16 (1.12-1.20)	1.15 (1.11-1.19)
Systolic blood pressure, per 10 mmHg	0.98 (0.96-1.01)	0.98 (0.95-1.01)	0.98 (0.96-1.01)
Oxygen saturation, per %	0.91 (0.89-0.92)	0.91 (0.90-0.92)	0.91 (0.90-0.92)
**GCS category:**			
15	Reference	—	Reference
14	2.17 (1.58-2.99)	—	1.63 (1.15-2.30)
13	3.09 (1.86-5.13)	—	1.51 (0.84-2.69)
9–12	7.22 (5.20-10.03)	—	2.91 (1.86-4.56)
3–8	11.44 (7.05-18.58)	—	3.64 (1.71-7.74)
**RASS category:**			
0	—	Reference	Reference
−1	—	2.31 (1.60-3.33)	1.78 (1.20-2.64)
−2	—	10.25 (6.50-16.17)	5.33 (3.11-9.15)
−3	—	7.26 (4.17-12.63)	2.81 (1.42-5.57)
≤−4	—	12.06 (7.51-19.35)	3.99 (1.91-8.32)
+1	—	4.83 (3.18-7.32)	4.67 (3.07-7.09)
≥+2	—	7.11 (3.97-12.72)	4.26 (2.27-8.03)
AUC (95% CI)	0.758 (0.739-0.778)	0.762 (0.742-0.781)	0.765 (0.745-0.784)

aOR: adjusted odds ratio; RASS: Richmond Agitation–Sedation Scale; GCS: Glasgow Coma Scale. The clinical model comprised age, sex, heart rate, systolic blood pressure, and oxygen saturation.

Likelihood-ratio tests: adding RASS to the clinical model $\chi^2=282.9$, df=6, $p<0.001$; adding GCS $\chi^2=218.3$, df=4, $p<0.001$; RASS beyond GCS $\chi^2=90.1$, df=6, $p<0.001$; GCS beyond RASS $\chi^2=25.5$, df=4, $p<0.001$.
